# Ultra-High-Performance Concrete (UHPC): A State-of-the-Art Review

**DOI:** 10.3390/ma15124131

**Published:** 2022-06-10

**Authors:** Rahat Ullah, Yuan Qiang, Jawad Ahmad, Nikolai Ivanovich Vatin, Mohammed A. El-Shorbagy

**Affiliations:** 1 School of Civil Engineering, Central South University, Changsha 410075, China; rahataryan90@gmail.com; 2 National Engineering Research Center of High-Speed Railway Construction Technology, Changsha 410075, China; 3 Department of Civil Engineering, Swedish College of Engineering and Technology, Wah Cantt 47040, Pakistan; 4 Peter the Great St. Petersburg Polytechnic University, 195251 St. Petersburg, Russia; vatin@mail.ru; 5 Department of Mathematics, College of Science and Humanities in Al-Kharj, Prince Sattam bin Abdulaziz University, Al-Kharj 11942, Saudi Arabia; ma.hassan@psau.edu.sa

**Keywords:** ultra-high-performance concrete, fibers, secondary cementitious materials, mechanical strength, durability aspects

## Abstract

The interest of researchers in UHPC has increased over the past decade. It is crucial to understand the structural behavior of reinforced UHPC (R/UHPC) components under various loading conditions before they can be used as a replacement for conventional concrete. Although several studies on ultra-high-performance concrete (UHPC) have been conducted, the knowledge is scattered, and no one can easily judge the performance and methodology of UPHC. Therefore, the purpose of this study was to review the research studies already carried out on UHPC. The review focuses on the materials’ physical and chemical composition, mechanical and durability characteristics, fire resistance, and environmental benefits of UHPC. Design considerations for effectively utilizing UHPC in structural elements are also presented. The best UHPFRC mixture is obtainable with a steel fiber content of 2–3% and a water-to-cement ratio of 0.2–0.3. The review also discusses the essentials recommendation for future research on UHPC.

## 1. Introduction

Conventional concrete is strong in compression and weak in tension, and fails when brittle without any deformation. Ultra-high-performance concrete (UHPC), which is a cutting-edge concrete, may be able to resolve these problems. According to the Federal Highway Administration (FHWA) of the United States, UHP cement is a cementitious material formulated by combining Portland cement, fine silica sand, silica fumes, quartz flour, high-range water reducers, and discontinuous internal combustion steel or organic fibers, and a water-to-cement ratio less than 0.25. The strength of UHPC is greater than 21.7 ksi (150 MPa) compressive strength and greater than 0.72 ksi (5 MPa) tensile strength sustained post-cracking [1]. Meng et al. [2] suggest that ultra-high-performance concrete (UHPC) is a new type of cementitious composite made with extremely low water-to-binder ratios. The water-to-binder ratio for UHPC ranges from 0.18 to 0.22. ACI 239 states that the compressive strength of UHPC is more than 150 MPa after 28 days [3]. In post-cracking testing, strain hardening behavior is observed in UHPC after being cracked at tensile strengths of 7–15 MPa [4]. To achieve highly flowable, high mechanical, and excellent durability, ultra-high-performance concrete (UHPC) should be produced using the optimum combination of cement and supplementary cementitious materials, adequate sand gradation, low water-to-binder ratio (w/b = 0.25), fiber reinforcement, and high-range water reducer (HRWR) [5]. UHPC offers better durability than conventional concrete because of its discontinuous pore structure [6].

Fibers can also provide UHPC with strain-hardening properties in tension and convert brittle failure to ductile failure [7]. Several studies have optimized composition, enhanced performance, reduced cost, and saved energy in manufacturing UHPC [8]. It is possible to transfer the stress between the fibers at the cracked section to the uncracked UHPC matrix by using the fiber–matrix bond [9]. It is important to understand that the efficacy of fiber–matrix bonds depends on the strength of the adhesive provided by the UHPC mortar in the region around the fibers [10]. It is important to optimize the fiber distribution as well. A higher degree of dispersion of the fibers by the UHPC mortar can lead to a higher degree of encapsulation of the fibers by the mortar to achieve higher fiber–matrix interfacial properties [11]. A recent study determined that even under four-point bending, the post-cracking behavior of UHPC exhibits strain hardening when the content of micro steel fibers exceeds 1% by volume of the UHPC. There was, however, a finding that the microfibers were ineffective at delaying the initiation and propagation of microcracks, which may be due to the relatively large spacing between the fibers [12]. A study observed that fibers improved tensile capacity of concrete more effectively than compressive strength [13]. Therefore, secondary cementitious materials (SCMs) play an important role in UHPC.

Silica fume is a SCM that serves as a critical component in the production of UHPC. It plays a significant role in affecting its overall performance because of its high amorphous SiO_2_ content and the ability to increase the packing density of the matrix. It has small, glassy, and spherical characteristics of a ball bearing that can create the ball-bearing effect and help replace water entrapped between the fine and coarse particles [14]. There can be a reduction in the mixture’s flow resistance (i.e., the viscosity), making it possible for the mixture to be well dispersed into multiple phases with reduced porosity and a dense microstructure [15]. As a consequence of SCMs’ consumption of calcium hydroxide (CH), it forms a strength-contributing substance used in making C-S-H gel. In this case, a substance was found to have similar behavior to that formed from Portland cement, thus improving the mechanical properties of concrete [16].

Furthermore, it can also improve the interfacial transition zone (ITZ) between aggregate/fiber and matrix to eliminate large preferentially oriented CH crystals, ultimately leading to improved homogeneity [17]. The optimum dose of silica fume is also important as the higher dose can cause alkali–silica reaction due to the dilution effect. Previous investigations have indicated the typical silica fume content used in UHPC mixture is approximately 20–30% by mass of cementitious materials [18,19].

As a result, UHPC is an ideal material to provide greater strength and durability to components or structures at the same time as it increases their strength and durability. However, knowledge is scattered and no one can easily judge the benefits of UHPC. Therefore, this review paper examines the mechanical properties, durability properties, and thermal properties of UHPCs. It also discusses the environmental and cost benefits of UHPC.

## 2. Materials

### 2.1. Cement

The choice of a brand and type of cement is probably the most important factor in selecting material for high-strength concrete mixtures. In the case of high-strength concrete, cement variation causes the concrete compressive strength to fluctuate more than any other single component. In fact, cement variation seemed to have a greater influence than any other individual concrete component. The cement should be selected based on its water demand; the normal consistency in the cement paste determines the water demand. It is observed that the highest water demand results in the lowest compressive strength. This cement also exhibits good workability and admixture compatibility, and does not possess false setting characteristics. Good quality cement is required to produce uniform high-strength concrete consistently [20].

### 2.2. Chemical Admixture

Polycarboxylate-based high-range water-reducing agent was used since ultra-high-strength concrete’s composition has a low water-binding material ratio. Polycarboxylate-based superplasticizers are suitable in the range of 2.0–3.5 wt.%, based on the amount of the binding material.

### 2.3. Aggregates

The shape, surface texture, and mineralogy composition of coarse aggregate must be considered when designing UHPC. The shape and surface texture of the aggregate, together with the mineralogy of the aggregate, affect the overall mixing water required for the concrete. Thus, they play a greater role in the strength-producing qualities.

#### 2.3.1. Coarse Aggregate

Cubical-shaped coarse aggregate is the preferred satisfying requirement of IS:383 [21]. The grading should satisfy the IS:383 [21] limit. The mineralogy of coarse aggregate granite, aplite, basalt, rhyolite, quartzite is recommended. Further, the crushing value should be below 17%. According to research, a smaller size aggregate produces higher compressive strength; the maximum particle size of the coarse aggregate should be set between 20 mm and 30 mm to achieve a higher compressive strength [13].

#### 2.3.2. Fine Aggregate

**Figure 1:**
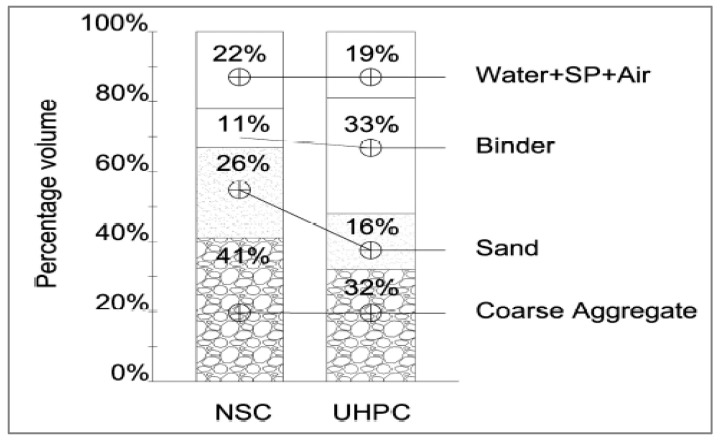
Aggregates for NHS and UHPC [23].

It is strongly recommended that the fine aggregate fineness modulus is at 2.8–3.0, as it assures the concrete’s derivable fluidity and reduces the viscosity of the final mix. It should satisfy the IS:383 [21] limit of the zone-II sands [22]. Natural sand produces a higher strength than the manufactured sand produced for either limestone or traprock. The advantage is attributed to reduced mixing water demand for less angular material. Further, a fineness modulus of 3.0 is desirable since increasing the fineness of either type of sand reduces compressive strength. The Figure 1 shows percentages of aggregate normal strength concrete (NHS) and UHPC.

### 2.4. Micro Silica/Silica Fume

Micro silica or silica fume are exceptionally fine micro particles of amorphous silica, which are included in concrete mixes. They are pozzolanic highly reactive silica, which increases the durability and strength of the concrete. The micro silica/silica fume should satisfy the requirements of ASTM C-1240 [24] and BS EN- 13,263 [25]. Minimum SiO_2_ (silicon dioxide) should be 85%; maximum C (carbon) allowed = 2.5%.

### 2.5. Steel Fibers

**Figure 2:**
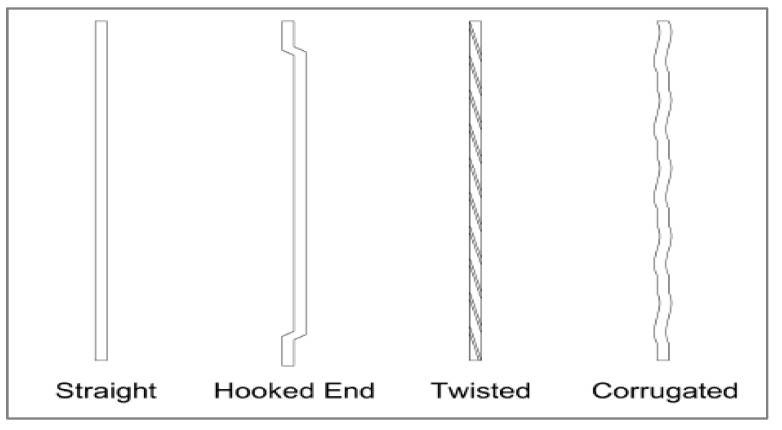
Shapes of fibers [23].

Due to its very high strength and homogeneity, UHPC is very brittle; yet it can be made ductile by adding steel fibers [8]. Fibers provide greater resistance to crack generation and propagation [13,26]. Figure 2 shows the shape of fibers, while Table 1 shows the different physical aspects of fiber used for UHPC as per past studies.

### 2.6. Mix Design

The mix design of UHPC should be economical and sustainable to achieve a denser matrix, reduce porosity, and improve the internal microstructure to produce superior mechanical and durability properties. The mixture design should also promote economics. The use of mixture designs for UHPC has been reported under various models. The main parameters considered within the initial design process to achieve an improved homogeneous microstructure with dense and ductile properties are optimization of granular mixtures, elimination of coarse aggregates, and proper integration of fibers with the granular mixtures [36]. Considering the shape, size, and density of particles, a researcher produces a UHPC mixture. There was also a report claiming that by using fine-grained multi-grain cement, particle sizes can be decreased [37]. UHPC mix design aims to eliminate the pore spaces of the final matrix from the micro cracks by eliminating defects in the design. An expanded particle packing model adapted from Andreasen and Andersen has been used to develop a densely compacted UHP with a cement content lower than 675 kg/m^3^ [38].

Furthermore, a few statistical models were proposed that could simulate the mixture design of UHPC. In the case of UHPC, an adaptive neuro-fuzzy interface system (ANFIS) was used for proportioning the ingredients of the mixture [39] to calculate the maximum flexural strength of self-compacting steel fiber-reinforced UHPC with varying contents of steel fiber [40]. A researcher also used a RSM model for optimization concrete properties [41,42,43]. Table 2 shows mix proportions of UHPC.

## 3. Fresh Properties

**Figure 3:**
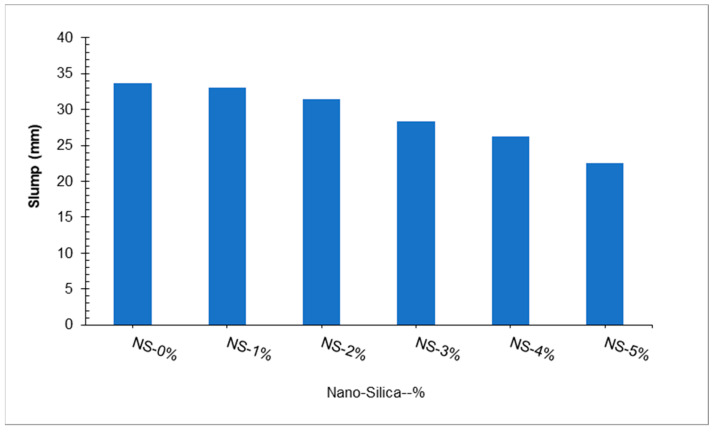
Slump flow [51].

Yu et al. [51] studied the behavior of flowability of UHPC. The UHPC was prepared with a constant 2.50% steel fiber and varying percentages of nano-silica in the proportion of 1%, 2%, 3%, 4%, and 5%. Superplasticizer (polycarboxylic ether) 4.5% was also kept constant to maintain workable concrete. Test results indicate that slump value decreased with the increased nano-silica substitution ratio, as shown in Figure 3. Maximum slump value was obtained at 0% substitution of nano-silica, while minimum slump was achieved at five substitutions of nano-silica. The decrease of slump value may be due to the pozzolanic reaction of nano-silica, which increased the viscosity of cement paste, leading to less slump. Moreover, it may be attributed to the increase in the specific surface area associated with an increase in fiber content [38]. The nano-SiO_2_ that is replaced with cement has a much higher reaction capacity, so it is easier to attract surrounding water molecules to form chemical bonds. The fast reaction of SiO_2_ can be attributed to its high specific surface area and many unsaturated bonds. Therefore, there is no water separation or obvious exudation of water from the nano-SiO_2_ mixture [52]. Further, the steel fibers were randomly distributed throughout the matrix and acted as a skeleton, preventing new concrete from flowing into the matrix [26]. A decrease in porosity was also observed with the substitution of nano-silica. However, at 5% substitution of nano-silica shows a slightly increased porosity due to lack of flowability, which increases the compaction afforded, leading to more voids in hardened concrete. In contrast, Wang et al. [53] made UHPC with steel fiber, ground granulated blast furnace slag, and silica fume. The water-to-binder ratio was kept constant (0.18). Results indicate that the flowability of UHPC increased with the increase in the substitution of ground granulated blast furnace slag. The shape of the fiber also affected the flowability of fiber. In terms of flowability, samples with hook-end fibers exhibited the lowest values compared to those with straight fibers and corrugated fibers. Compared to a mixture with the same number of straight fibers, mixtures with 1%, 2%, and 3% hooked-end steel fibers exhibited reduced flowability of 20.9%, 35.8%, and 51.2%, respectively. There were reductions of 17.7%, 31.2%, and 45.1%, respectively, for the mixtures with 1%, 2%, and 3% corrugated fibers [27]. As a result, deformed fibers could increase the friction between fibers and aggregates, thereby increasing cohesion with the matrix and, therefore, reducing the flowability [26]. Besides, a change in fiber shape can lead to a strengthening effect among fibers, which tends to make fibers bundle together more easily [38]. Table 3 shows a summary of the fresh properties of UHPC.

## 4. Mechanical Properties

### 4.1. Compressive Strength

**Figure 4:**
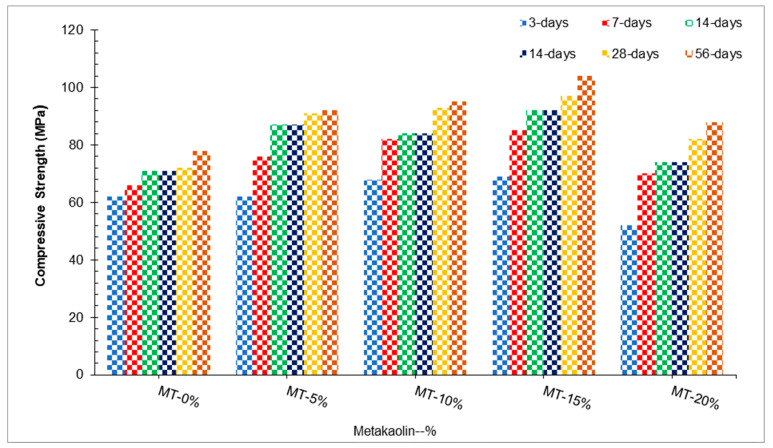
Compressive strength [55].

Mo et al. [55] used 0–20% metakaolin (MT) in increments of 5% while keeping a constant 30% limestone and 0.2 water-to-binder ratio to prepare UHPC. Test results indicated that maximum compressive strength was achieved at 15% (optimum) substitution of MK by weight of cement as compared to the reference concrete, as shown in Figure 4.

A study also reported that maximum compressive strength was obtained at 15% substitution of metakaolin [57]. This reduction is attributed to a clinker dilution effect that is responsible for reducing the compressive strength of 20% metakaolin. This is because the equivalent quantity of metakaolin replaces a part of the cement. The filler effect and the pozzolanic reaction of metakaolin with calcium hydroxide result in an increase of the compressive strength of concrete [58]. Because of this very reason, an optimum replacement for metakaolin is recommended in concrete. It is expected that the differences in compressive strength between the metakaolin mixtures and the OPC concrete will become smaller with time. The possible explanation for this might be that all cementitious materials’ reactions had now finished or had stopped because the reactions between the metakaolin and OPC mixtures had slowed down over time [59]. It was found that the compressive strength of specimens containing metakaolin increased with time. Still, when specimens containing 25% of metakaolin were examined, the strength characteristics did not match those of standard mortar specimens. When metakaolin is incorporated into cement-based composites, the result is an increase in compressive strength attributed to its filler effect in the zone of interfacial transition between the cement paste and aggregate particles.

Additionally, CH gels are removed during the hydration of cement with metakaolin and are actually responsible for accelerating the cement–cementitious hydration process [60]. Yu et al. [51] carried out research on UHPC concrete with 2.5% steel fibers and 4.5% superplasticizers with varying percentages of nano-silica. Results indicate that a maximum compressive strength (91 MPa) was achieved at 4% nano-silica, which was 17% higher than that of reference concrete (78 MPa). A major factor that improves concrete compressive strength is the pozzolanic reaction between nano-SiO_2_ and calcium hydroxide, which promotes the formation of hydrated calcium silicate. Concrete without nano-silica can only hydrate to a very small amount of calcium silicate hydrate if the cement does not contain nano-silica. Calcium silicate hydrate is one of the vital elements that provide strength to concrete. Because of this, concrete without nano-SiO_2_ has a low compressive strength [61]. According to a study, the early strength improvement effect of nano-silica-modified concrete is more evident, and this is because of the higher pozzolan activity of nano-SiO_2_ particles [62]. It is important to note that with the prolongation of the curing time, the concentration of nano-silica particles used for the pozzolanic reaction gradually declines, thus reducing the compression improvement effect of the later-stage of nano-silica modified concrete [63]. Research was carried out on the compressive strength of UHPC produced with fiber volumes ranging from 0–3% and waste glass concentrations ranging from 0–0.27%. Increasing the fiber volume of UHPC enhanced the compressive strength of the material over a 28-day period at a fixed waste glass content due to a rise in the elastic modulus [64] as well as the steel fiber’s capacity to bridge tiny cracks [65]. The results of the compressive strength tests improved when the quantity of silica fume was raised from 10% to 25% but there was no statistically significant difference between 25% and 30% replacement [46]. The 3-day compressive strength rose gradually in response to an increase in temperature, reaching 181 and 229 MPa, respectively, at 140 and 200 degrees Celsius [46]. According to the findings of the research, ultra-fine fly ash with a mean particle size of 4.48 microns demonstrated its suitability for use in UHPC with a 20 weight percentage cement replacement, resulting in a paste with a compressive strength of 153 MPa [66]. Table 4 shows a summary of the mechanical performance of UHPC.

**Figure 5:**
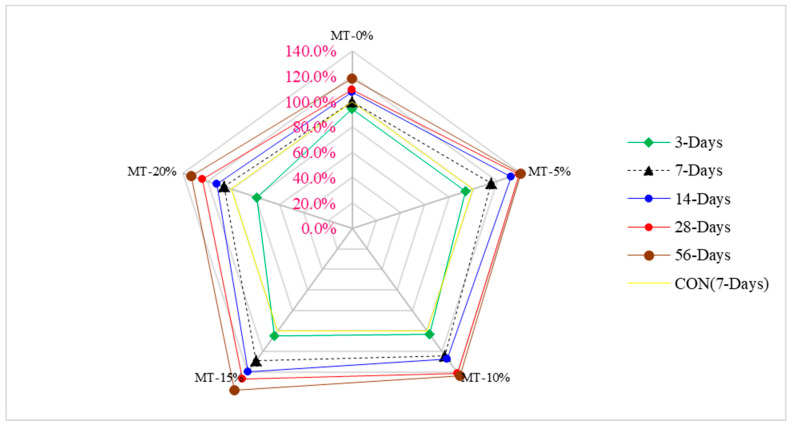
Compressive strength–age relationship: data source [55].

Figure 5 shows the strength–age relationship of compressive strength in which 7 days’ control compressive strength of UHPC was considered as reference strength. The optimum dose of metakaolin (15%) was considered for comparison with days of curing. At 3 days of curing, compressive strength of 15% metakaolin is approximately equal to that of the reference concrete (7 days’ control concrete compressive strength). Compressive strength at 7 days’ curing at 15% metakaolin is 28% more than that of reference concrete, while 14 days’ compressive strength of UHPC is 39% more than that of reference concrete. At 28 days of curing, the compressive strength of UHPC is 47% more than that of reference concrete. At the same dose (optimum dose), the compressive strength of UHPC is 58% more than that of reference concrete at 56 days of curing.

### 4.2. Flexure Strength

**Figure 6:**
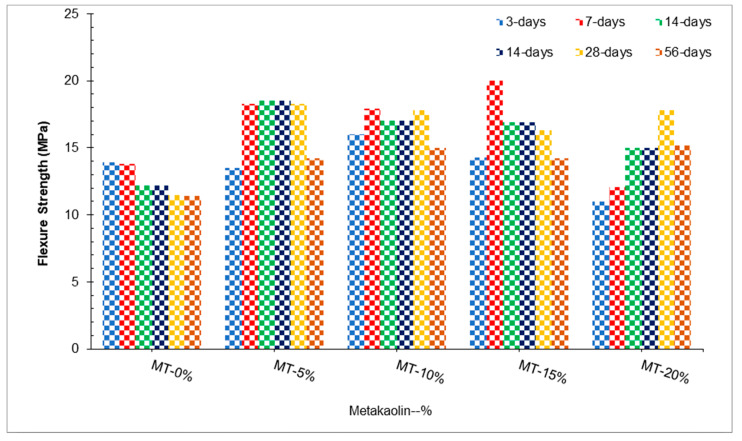
Flexure strength [55].

The inclusion of metakaolin in cement-based composites enhances compressive strength through the filler effect in the interfacial transition zone between the cement paste and aggregate particles. In addition, CH gels are quickly removed during the hydration of cement with metakaolin and actually accelerate cementitious hydration [54]. Mo et al. [47] concluded that similar to the compressive strength, maximum flexure strength was achieved at 15% substitution of metakaolin. In comparison, minimum flexure strength was obtained with 0% substitution of metakaolin, as shown in Figure 6. Based on the results obtained, the 7-day flexural strength increases as the metakaolin content increases, which can be explained by the production of C–S–H from pozzolanic reactions and reduction of chloride attack through the development of Friedel’s salt [67]. In contrast, a study found that MK incorporation decreases flexural strength. The decreased in flexure strength could be attributed to the low w/b ratio used in addition to the high absorption capacity of metakaolin particles provided by the surface roughness [68]. Table 4 shows a summary of the mechanical performance of UHPC.

**Figure 7:**
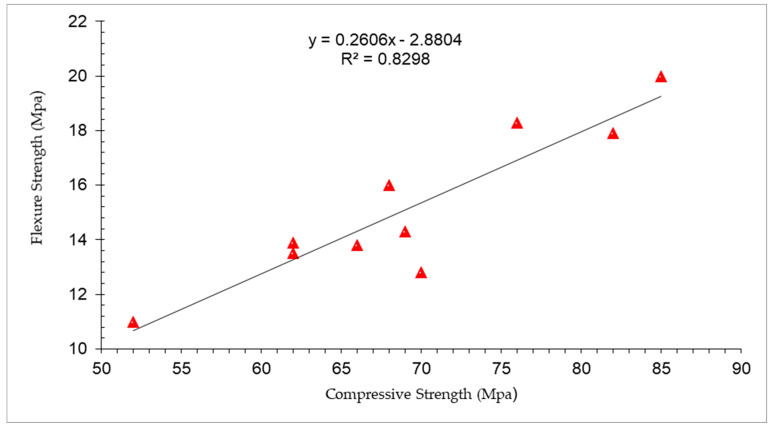
Correlation between compressive and flexure strength: data source [55].

Figure 7 shows the correlation between compressive and flexure strength at 3 and 7 days of curing. It can be noted that a strong correlation exists between compressive and flexure strength having a R^2^ value approximately equal to 90% (83%).

**Figure 8:**
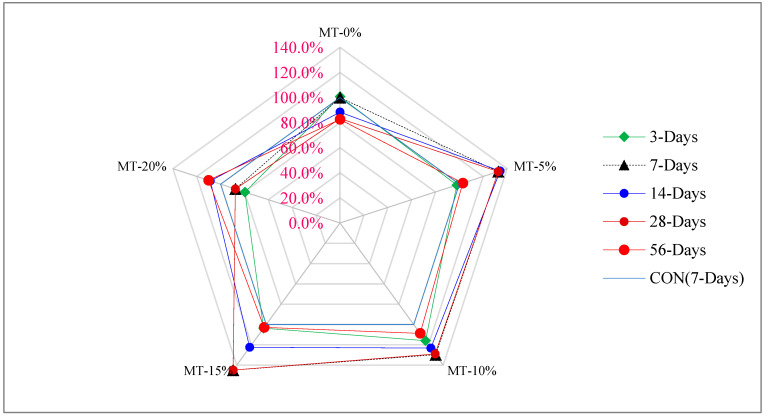
Flexure strength–age relationship: data source [55].

Figure 8 shows the strength–age relationship of flexure strength in which 7 days’ control flexure strength of UHPC was considered as reference strength. The optimum dose of metakaolin (15%) was considered for comparison with days of curing. At 3 days of curing, flexure strength of 15% metakaolin is approximately equal to that of the reference concrete (7 days’ control concrete compressive strength), similar to the compressive strength. Flexure strength at 7 days’ curing at 15% metakaolin is 44% more than that of reference concrete, while 14 days’ flexure strength of UHPC is 23% more than that of reference concrete. At 28 days of curing, the compressive strength of UHPC is 45% more than that of reference concrete. It can be concluded that flexure of UHPC is much higher than its compressive strength due to the presence of fibers.

**Table 4:** Summary of mechanical performance of UHPC.

Authors/ Reference	Material	W/C	Fiber Type %	Compressive Strength (MPa)	Flexural Strength (MPa)	Tensile Strength (MPa)
Wu et al. [27]	Silica fume	0.18	Straight 0 1 2 3 Corrugated 1 2 3 Hooked 1 2 3	----- 110 125 145 150 ------- 135 145 155 ------ 140 155 165	---- 18 20 25 35 ---- 23 28 37 ---- 25 32 40	------ ------ ------ ------ ------ ------ ------ ------ ------ ------ ------ ------- -------
Shafieifar et al. (2017) [28]	Premix-ductal	0.15	Straight 0% 2%	----- 40.4 138	8.3 37.6	4.9 21.9
Zemei et al. [29]	Silica fume 0 5 10 15 20 25	0.18	2%	----- 81 98 112 115 113 110	------ 13.06 14.38 17.23 14.63 15.15 13.84	------ 4.53 5.23 7.87 7.65 6.01 5.76
Wang et al. [53]	SF 10% GGBS 0 20 40 LP 0 20 40	0.18	NA	138 122 110 142 150 130	--- --- --- --- --- ---	--- --- --- --- --- ---
W. Meng et al. [30]	GNPs SF FA	0.2	%GNPs/%CN’s 0/0 0.05/0.05 0.1/0.1 0.15/0.15 0.2/0.2 0.3/0.3	GNPs/CNFs 174/174 174.5/16 177/177 180/178 182/181 184/184	GNPs/CNFs 7.73/7.73 8.17/8.49 8.28/8.94 10.7/9.53 11.12/10.1 11.26/10.7	GNPs/CNFs 5.84/5.84 7.01/6.49 7.65/6.99 7.97/7.32 8.36/7.67 9.09/8.17
P. R. Prem et al. [44]	SF QP	0.2	SF 0 2 2.5	----- 132 175.28 171.35	----- 16 43 33.35	----- 11.3 23.2 19.1
Mo et al. [55]	LS 30% MK 0 5 10 15 20	0.2	NA	---- ---- 72 91 93 97 82	---- ---- 11.5 18.3 17.8 16.3 17.8	---- ---- ---- ---- ---- ---- ----
C.C. Hung et al. [54]	SF + QP	0.135	Macro-steel fiber 0 1 2	---- 150 126 132	--- --- --- ---	--- 5.9 6.1 6.6
Aziz and Ahmed [28]	SF	0.16/0.62 0.62 0.16 0.16 0.2	SF 0 0 0.12 0.26	---- 38.8 131.4 125.2 126.8	---- 3.95 22.24 18.45 19.63	--- 2.97 11.03 8.91 13.76
M.A. Ibrahim et al. [46]	SF (10%, 20%, 30%) GGBS (10%, 20%) FA (10%, 20%) GS (0%, 5%, 10%, 15%, 20%, 25%)	0.18 to 0.24	SF + GS	159 139 146 158 ----- 144 152 154 155 160 161	104 132 144 160 159 160 144 154 147 ---- ----	------ ------ ------ ------ ------ ------ ------ ------ ------ ------ ------
			SF0 SF10 SF20 SF30 GS 0% 5% 10% 15% 20% 25%			
H.J. Chen et al. [49]	Silica fume-SF Ultra-fine silicon powder-SFP	0.195	St. fiber	(St.F + PPF + SF + SFP)%	90 128.1 127 ------ 121.6 123.6 125.8	12.7 6.6 12.6 ------ 14.7 12.2 12.7
			0.5 0.75 1 PP-fibers 0.03 0.06 0.09	0 + 0 + 0 + 0 0.5 + 0.03 + 17.4 + 2.2 1 + 0.06 + 18.7 + 2.2 ----------------- 0.75 + 0.03 + 18.7 + 3.2 0.75 + 0.09 + 20 + 2.2 1 + 0.03 + 20 + 2.7		
Yu et al. [51]	Nano-silica 0 1 2 3 4 5 ---- 0 1 2 3 4 5	0.4	Macro-steel fiber 0% 2.5%	---- --- 78 79.9 81.5 89.2 91.3 86.9 ----- 113 120 129 136 138.4 136.4	--- --- 10.4 11.9 12.8 13.4 14 13.2 ------ 18 21.2 22.5 24.4 25 24.1	--- ---
Teng et al. [45]	Welan Gun Powder-WG (0%, 0.18%, 0.22%, 0.27%) Class-c fly ash- 40% Silica fume 5% Air-detraining admixture, polyether, 0.8%	0.2	Straight steel fibers 0%	115 112 110 105 --- 120 119.8 119.5 117 --- 128 127 125 121 --- 133 132 131.2 130	9 9 8 7 -- 15 17 16 15 --- 16 19 21 20 --- 20 23 26 27	
			WG–0% WG–0.18% WG–0.22% WG–0.27%			
			1%			
			WG–0% WG–0.18% WG–0.22% WG–0.27%			
			2%			
			WG–0% WG–0.18% WG–0.22% WG–0.27%			
			3%			
			WG–0% WG–0.18% WG–0.22% WG–0.27%			
Azmee et al. [32]	SF%–FA% 0–0 0–30 0–40 0–50 10–30 10–40 10–50	0.16	Steel fiber = 1%	120 127 120 9898 128 130 117	------ ------ ------ ------ ------ ------ ------	------ ------ ------ ------ ------ ------ ------
Kwon et al. [69]	SF Anti-foaming agent	0.22	Micro SF (const)-straight = 1% Macro SF (varying) 0.5 1 1.5 2	28-day avg compressive strength = 182 MPa	11.9 12.4 16.1 20.1	------ ------ ------ ------

## 5. Durability

### 5.1. Water Absorption and Porosity

It is well known that concrete with high water permeability can become a barrier that allows chemicals, such as chloride ions, to diffuse into it and eventually result in corrosion of steel rebars and/or fibers. UHPC is characterized by much smaller porosity and a much denser microstructure than both conventional concrete (CC) and high-performance concrete (HPC). The low porosity makes UHPC a superior permeability-resistant material. The water absorption capability of concrete can easily provide information on factors such as the porosity and quantity of permeable pores and the interconnectedness of those pores [70]. The durability of concrete increases with a decrease in the water absorption capacity of the concrete.

In comparison to HPC, UHPC’s potential absorption of water is about ten times lower, and it is 60 times lower than NSC’s potential absorption of water [71]. The reduction of pores in UHPC means UHPC has excellent durability [72]. A study found that the pores have an average diameter less than 5 nm and the volume of the pores is between 1% and 2% of the total volume of the pores in UHPC [73]. It has been found that the water absorption coefficient of UHPC after 90 days is approximately five times lower than that of control concrete [74]. An analysis of one study revealed an average water penetration height and a relative seepage height of 7.2 and 2.2 × 10^−8^ mm, respectively, as determined by a single pressure method [75]. Compared to the reference mixture, the UHPC-NSC made with nanoparticles showed a 36% lower water absorption rate than the reference mixture. The gas permeability coefficient of UHPC is less than 1.0 × 10^−19^, which is three orders of magnitude lower than the gas permeability coefficient of conventional concrete. When the porosity of the pores is low, and the pore connectivity is restricted, water absorption is greatly reduced. With the addition of mineral admixture to UHPC, the microstructure of UHPC becomes more homogeneous, and the thickness of the ITZ is significantly reduced. In such a case, it reduces the UHPC’s water absorption capacity because it partially blocks its water transport pathway [76].

### 5.2. Chloride Penetration

It has been identified that chloride ion penetration resistance is one of the most critical factors in concrete strength. It is known that concrete that has a higher tolerance to chloride has a higher ductility. A series of variables such as the w/b ratio, exposure conditions, curing regime, and exposure duration determine the degree of chloride penetration [77]. Chloride ions diffuse in the concrete’s pores or can be chemically and physically bound to hydration products [78]. It is also possible to estimate the penetration of chloride ions by using the rapid chloride ion penetrability test in terms of the number of coulombs (electric current) passed through the specimens [79]. Chloride can be classified into free chlorides and bound chlorides, depending on the binding method. Chloride ions are chemically bound to cement compounds and can react with their compositions to form salts. A penetration of free chloride ions into steel/fiber-reinforced concrete may result in passivation of the steel rebar and/or fiber and the initiation of a corrosion process leading to the degradation of concrete structures [80]. A high alkaline pore solution is used for passivating steel reinforcements in concrete to protect against corrosion. It has been found that the passive layer on the steel surface can be damaged by aggressive chloride ions and/or the neutralization of the environment near the reinforcements. This corrosion leads to the deterioration of steel reinforcements and eventually to the deterioration of concrete structures, reducing their service life [81]. It has been concluded from research that the distribution and the interconnection of pores and cracks are important factors contributing to the water transport and distribution in cement-based materials, which have a significant impact on their permeability. Based on previous studies, it has been shown that the filling effect and the nucleation effect of NS can significantly improve the compactness of concrete by boosting cement hydration [82]. A study noted that the chloride ion diffusion coefficients of UHPC were less than 1.4 × 10^–13^ m^2^/s [83]. A study noted that the addition of steel fibers to UHPC did not cause any electrical short-circuiting during the rapid chloride ion penetration test because they were shorter in length and randomly distributed throughout the material [84]. It was also found that the total value of charges passed through thermally treated UHPC specimens was 22 coulombs, which is much lower than the values for HPC (216 coulombs) and NSC (1736 coulombs) [85].

### 5.3. Freezing and Thawing

The damage caused by freezing–thawing occurs in concrete when water molecules freeze and expand beyond the volume limitations of the concrete. As a result, concrete becomes distressed, especially when the pressure develops higher than its tensile strength, eventually resulting in dilation and rupture of the cavities within. In most cases, concrete deterioration is caused by freeze–thaw action, including random cracking, surface scaling, and joint deterioration due to cracking [86]. It has a negative effect on both the mechanical and permeability properties of the concrete and the other durability properties of the concrete. It is especially difficult for the UHPC to deal with freezing and thawing. This factor is essential for achieving a microstructure with an improved degree of homogeneity, a lower permeability, and a reduced porosity [87]. It is generally possible to maintain 400–500 freezing–thawing cycles and 4500 wetting–drying cycles without deteriorating the material [88]. The reduced porosity and permeability of UHPC allow it to resist freezing and thawing more effectively [89]. A freeze–thaw degradation was not observed in UHPC specimens after 800 freezing–thawing cycles, which was attributed to fewer interconnected pores between the specimens [90]. Concrete frost resistance is generally thought to be impacted by a number of pore structure parameters, particularly the amount of porosity, the average diameter of air voids, the distribution of pore size, air content, and the spacing coefficient between air voids [91]. A study found no considerable deterioration on UHPC after 500 freezing–thawing cycles along with 4500 wetting–drying cycles. A further observation was made to show that the addition of steel fibers appears to decrease the degradation of the material inside by freezing and thawing [92]. With just 10% more fly ash and 10% more silica fume, the UHPC’s resistance capacity is significantly improved [93].

### 5.4. Dry Shrinkage

Concrete shrinks when it dries out, which leads to cracking, which is caused by the loss of water due to evaporation. The low w/c ratio facilitates the self-desiccation of UHPFRCs. In the self-desiccation process of concrete, the internal relative humidity of the concrete decreases during the hydration process, thus reducing the size of the pores and resulting in increased capillary tension inside each individual pore. The presence of self-desiccation condition induces the rapid development of the autogenous shrinkage of UHPFRC at an early stage. However, only a very small amount of moisture exchange occurs in the environment due to this condition. As a result, no shrinkage occurs after drying. However, a substantial part of shrinkage occurs within the first few minutes after setting [94]. There are several types of shrinkage in concrete, including chemical, carbonation, mechanical, autogenous, and thermal shrinkage. According to a researcher, these types (autogenous and thermal) of shrinkage are common in concrete. In addition, UHPC’s low porosity and the evaporation of internal water are low, which minimizes drying shrinkage [8]. However, the autogenous shrinkage of UHPC is a problem due to the high amount of cement consumption and the relatively low weight-to-volume ratio (w/b). Autogenous shrinkage is a term used to describe a decrease in the volume of cement components at the macroscopic level due to cement hydration after the first environment of setting [8]. A primary cause of this problem is the development of surface tension in the very fine capillaries of the concrete matrix, which is caused by an insufficient amount of water in the concrete matrix for the binder material to fully hydrate [95]. A study found that the shrinkage in the mixture of UHPC without steel fibers was approximately 135% more than that of the mixture with 2% steel fibers. Shrinkage reduction is explained by the fact that as the cementitious matrix shrinks, shear stresses appear along the fiber matrix interface [96]. In response to these stresses, fibers are compressed, causing them to resist the tensile strains in the matrix because of shrinkage. Table 5 shows a summary of the durability performance of UHPC.

### 5.5. Creep of UHPC

Creep is the propensity of a substance to permanently distort under sustain load. Concrete produces an instantaneous elastic strain when compressed [96]. A lower strain rate allows for increased creep and crucial crack propagation, leading to greater defects. In addition to the loading situation, concrete’s sensitivity to strain rate varies. Concrete creep is influenced by several factors such loading rate, specimen size and shape, humidity, and sustain stress [97]. UHPC exhibit high immediate and time-dependent deformations under compression and tension when loaded at early ages. This is due to the comparatively low stiffness of the material at an early age [98]. In the case of UHPC, the viscous character of the cement matrix is consolidated over time as a result of the lower water/cement ratio, while the water migrates into the concrete structure. In addition, the creep in the case of UHPC is greatly reduced once heat treatment is applied [99]. A researcher investigates the tensile and compression creep of ultra-high-performance concrete (UHPC), with and without steel fiber reinforcing. One of the most important findings of his research was that the tensile creep coefficient was equal to the compressive creep coefficient when cylindrical and prismatic samples were loaded at 50% of their strength [98]. A study found that thermally treated UHPC samples that were then subjected to long-term compression or tensile efforts had less creep (about 40%) than samples that were not thermally treated [96]. Another study found that raising the load had no effect on creep. The creep coefficient increased on cylinders exposed to long-term compression, even while the applied load was doubled [100]. According to the results of research, the steel fibers enhance the stiffness of the cross-sectional area. The steel fibers greatly shorten the amount of time required for the stresses to stabilize [101].

### 5.6. Density

**Figure 9:**
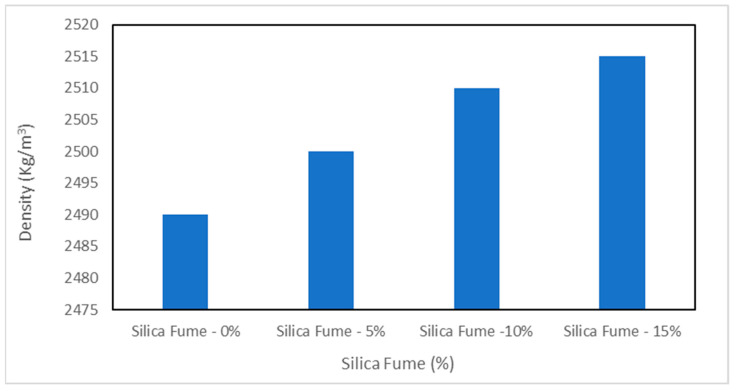
Density of UHPC [102].

The density of concrete also plays a vital role in its durability. A higher density of concrete results in more durable concrete as the water or harmful chemicals cannot penetrate into the concrete. Generally, UHPC has a higher density as compared to conventional concrete. The typical range of UHPC density is 2400 to 2500 kg/m^3^. Figure 9 shows the density of UHPC concrete. It can be noted that the density of UHPC increases with silica fume.

The increase in density for the 15% silica fume mix is about 1% larger than that of the 0% mix. The increase in density with silica is due to micro filling and the pozzolanic reaction; micro filling the voids in concrete ingredients leads to more dense concrete while the pozzolanic reaction of silica fume form secondary cementitious materials, i.e., calcium silicate hydrate, improves the binding properties of cement paste, leading to more dense concrete. Furthermore, UHPC concrete typical contains fibers, which also play an important role in the density of concrete. A study reported that the density of concrete increased with fiber. The increase in density of concrete due to addition of fibers can be attributed to crack prevention. Fiber prevents the propagation of cracks, resulting in more dense concrete [26]. Another study also reported that fibers restrict the development of dry shrinkage cracks, which improves the density of concrete. However, a further study claims that a higher proportion of fibers causes a decrease in density of concrete due to lack of flowability, which increases compaction, leading to more voids in concrete [13]. Therefore, the optimum amount of fiber is an important aspect of high-density concrete. Most researchers report 1–2% fiber as the optimum proportion.

## 6. Thermal Properties of UHPC

A major problem for ultra-high-performance concrete is the presence of fire or high temperatures (UHPC). After being exposed to 800 degrees Celsius, UHPCs may suffer a strength loss of up to 80% [103]. UHPC structures are more vulnerable to fire and elevated temperature due to the dense and compacted microstructure, low water-to-binder ratio, and reduced porosity. As a result, these structures can be damaged and harmed easily by the action of high temperatures and fire [104]. By exposing UHPC to a temperature ranging from 1000–1200 °C, physical and chemical changes occurred in the UHPC matrix, resulting in disintegration mainly due to these alterations. The main reason for disintegration in UHPC structures is exposure to final elevated temperature, blasting, and previous exposure to fire [105]. The literature shows that the addition of polypropylene (PP) fibers may help in controlling this problem and has the ability to lessen this issue. The fire resistance capacity of UHPC can be enhanced by the addition of 0.6% polypropylene fibers, which also results in preventing the spalling of concrete [72]. When the polypropylene fiber melts down at high temperature, it produces spaces to evacuate the accumulated pressure and so the fire resistance of the UHPC matrix is increased. Similarly, elevated temperature can also affect the compressive strength of UHPC. Studies showed that when the UHPC specimens were exposed to a temperature of 300 °C, the compressive strength increased but beyond 300 °C the strength was reduced [106]. This decrease in compressive strength is due to the weakened internal microstructure of UHPC because of high temperature [107]. According to the findings of the study, when compared to standard normal strength concrete beams, UHPC beams may have a poorer fire resistance [108]. Steel slag has a critical role in increasing the fire resistance of the UHPC structure [103]. UHPC is much denser than conventional concrete (due to its lower water/powder ratio and the absence of entrained air), so its thermal conductivity is higher than that of conventional concrete. This means that a high temperature in the concrete will be reached much sooner in UHPC than in conventional concrete. A study also observed that the thermal conductivity of UHPC is higher than that of conventional concrete [109]. The specific heat capacity of UHPCs is often lower than that of conventional PCs (again a denser material and with lower moisture content). Due to the fact that UHPC is often used in extremely thin elements, the thermal capacity of the element is reduced even more since there is less mass to absorb the heat. UHPC is not recommended for use in high-temperature applications. Similarly, another study reported that the specific heat capacity of UHPC is slightly lower than that of conventional concrete [109].

**Table 5:** Summary of durability performance of UHPC.

Authors/ Reference	Material		W/C	Fiber Type (%)		Water Absorption (%)		Chloride Penetration (Coulombs)	Freezing and Thawing- 28-Cycle (g/m^2^)		Porosity (%)
Alkaysi et al. [110]	SF = 25% White cement 0 15 25 Silica powder 0 15 25 GGBS 0 15 25		0.22	1.5%		-----		637 295 89 --- 57 488.5 939.5 ----- 137.5 229 137.5	17.7 20.7 98.8 ---- 42.2 18 18.2 ---- 44.7 24.2 20.5		-----
Ghafari et al. [111]	SF = 27% Nano-silica 0% 1% 2% 3% 4%		0.2	NA		1.2 1.1 0.95 0.80 0.85		6.35 4.74 4.66 4.30 4.80	-----		6.35 4.74 4.66 4.30 4.80
Abbas et al. [73]	Silica fume Quartz sand Quartz powder		0.23	Steel fibers 8 mm 1% 3% 6% 12 mm 1% 3% 6%		0.0589 0.0540 0.0477 -------- 0.0591 0.0544 0.0479		71 60 45 ---- 60 47 38			3.7 3.3 3 1.4 ---- 3.4 3.1 3.5
Scheydt et al. [112]	Silica fume quartz sand Quartz powder Basalt		0.21	SF = 2% 0 2%-(N-Tem) 2%-(90 °C)							8.9 10.9 5.4
Teichmann et al. [113]	Silica fume Quartz powder Quartz sand		0.5 0.33 0.24 0.17	SF %	OPC-kg/m^3^						15 8.3 6.2 2
				0 0 0 2	350 450 733 1000						
Piérard et al. [114]	Q-Powder SF-kg/m^3^ 100 166 156		0.23	Steel fiber 0 1 0							0 6 5
Huang et al. [115]	SF Kg/m^3^	RHA Kg/m^3^	0.2	NA				18 8 3.7 6 6.8 7.5	----- ----- -----		3.75 ---- 3.55 ----- 3.41 3.61
	276 230 184 138 92 46 0	0 46 92 138 184 230 276									
Coutinho et al. [116]	Silica fume 0% 10% Rice husk ash 10% 15% 20%		0.43	NA				2349.3 464.3 ------ 435 322 260			
Valipour et al. [117]	-Lightweight sand-(LWS) -CaO-based expansive agent (EXC) - MgO-based expansive agent (EXM) shrinkage reducing agent-SRA GGBS-50% LWS-50% EXC-7.5% EXC7.5%LWS25% EXC7.5%LWS40% EXC7.5%LWS60% EXC5%LWS60% EXC10%LWS60% EXM5%LWS60% EXM7%LWS60% SRA1.5%LWS60% SRA3%LWS60%		0.4	SF = 2%		Total shrinkage under initially air dried (AD), 3-day moist curing (3MC), and 7-day moist curing conditions (− reduction and + increased)					
						28 days			91 days		
						AD	3MC	7MC	AD	3MC	7MC
						−782 −780 −517 −623 −430 −513 −570 −94 −730 −690 −565 −318	−780 −698 −413 −519 −333 −400 −500 −26 −690 −528 −550 −280	−728 −580 −350 −460 −289 −273 −371 +254 −560 −480 −468 −250	−810 −830 −580 −680 −465 −535 −600 −110 −815 −756 −680 −455	−820 −750 −465 −575 −370 −453 −551 −30 −750 −610 −750 −366	−730 −630 −410 −470 −350 −321 −415 +105 −600 −520 −550 −310

## 7. Environmental and Cost Estimations of UHPC

Generally, the preliminary material cost of UHPC is greater than that of normal strength concrete and high-strength concrete because of its extremely high cement content and steel fiber addition. As a consequence of the use of UHPC, more sustainable building may be achieved, with potentially superior economic, social, and environmental benefits. Construction costs are closely related to the cross-sectional dimensions of structural parts. The usage of UHPC structural parts aids in the reduction of cross-sectional dimensions, hence allowing for the creation of more usable space in structures [118]. As a consequence of UHPC’s high strength, it is possible to build more thin structures, which results in a decrease in the building’s self-weight as fewer materials are used to form the structure. This may result in a reduction in demolition trash, which in turn reduces the need for transportation services; also, the transportation and labor cost of concrete decreases due to a lower quantity of materials used even if the cement content needed in UHPC is more than that required in conventional concrete. The cross-section size is bigger in the case of conventional concrete, which increases its cost. Therefore, the material cost of UHPC may be lower when compared to that of conventional concrete due to the larger cross-section. An investigation has shown that the usage of UHPC results in a 50% reduction in energy consumption when compared to the use of normal concrete construction methods [119]. Furthermore, the amount of fine particles can be reduced to 30%, while no coarse materials are used in UHPC [120]. Furthermore, the use of by-products such as fly ash and silica fume in place of cement makes UHPC more environmentally friendly and sustainable [84]. Because of the better durability of UHPC, UHPC needs less maintenance and as a result, life-cycle costs may be decreased [121]. Overall, because of its enhanced durability, environmental concerns and economic advantages, UHPC has the potential to be an environmentally friendly material.

## 8. Conclusions

One of the most important factors in producing UHPC is to improve mechanical and durability performance. A detailed review of the literature regarding the distinctive features of UHPC was conducted in this study. The following conclusions can be drawn based on the summary and discussion.

- UHPC should contain only fine aggregates like natural sand, silica sand, recycled glass cullet, quartz sand, etc., and not coarse aggregates because they will weaken the ITZ.
- UHPC normally exhibits lower workability as compared to normal strength concrete. UHPC normally contains fiber, increasing the internal friction between concrete ingredients, leading to lower workability.
- The best mechanical characteristics were obtained for UHPFRC mixtures when the water-to-binder ratio was less than 0.20.
- Maximum mechanical and durability performance was achieved at 2.0% addition of steel fiber by volume and hook-type fiber, further enhancing the performance of UHPC.
- Low water absorption, porosity, carbonation depth, freezing and thawing action, and dry shrinkage of UHPC make it usable in all types of aggressive environments.
- Thermal conductivity of UHPC is higher than that of conventional concrete due to higher density.
- UHPC is not recommended for use in high-temperature applications due to its low heat absorption capacity.

Finally, the overall performance of UHPC depends on the optimum percentages of each ingredient. Most researchers focus on the quality of UHPC ingredients, while a limited number of researchers have focused on optimization by performing statistical analysis such as using the response surface methodology or artificial neural networks for optimization of UHPC ingredients. Therefore, further study is recommended to optimize UHPC ingredients using statistical tools. Furthermore, limited studies are available on the durability aspects of UHPC.

## Figures and Tables

**Table 1:** Physical aspects of fibers.

Reference	Fiber Type	Length (mm)	Diameter (mm)	Tensile Strength (MPa)	Elastic Modulus (GPa)
Wu et al. (a) [27]	Straight Hooked-end Corrugated	13	0.2	2800	300
Shafieifar et al. [28]	Straight steel fiber	12.5	0.2	2600	278
Wu et al. (b) [29]	Straight brass-coated steel fibers	13	0.2	1900	203
Meng et al. [30]	Straight steel fiber Carbon nanofibers Graphite nanoplates Graphite nanoplates	13 50–200 × 10^3^ 25 30	0.2 100 2–10 2–10	1900 30,000 5000 5000	203 240 1000 1000
Kim et al. [31]	Brass-coated smooth steel fibers	19.5	0.2	2450	203
Azmee et al. [32]	Straight steel fiber Hooked-end steel fiber	20 25	0.2 0.3	>2300 >2300	>246 >246
Park et al. [33]	Straight fiber Hooked-end fiber	6 30	60 × 10^−3^ 380 × 10^−3^	2000 3000	206 206
Meszoly et al. [34]	Steel fibers	15	0.2	>2000	200
Christ et al. [35]	Steel fibers Polypropylene	13 6	0.21 0.12	2750 500–700	200 5

**Table 2:** Mix proportions.

Reference	Material	Utilization (Kg/m^3^)
Prem et al. [44]	Cement Silica fume Quartz W/C Steel fiber 13 mm × 0.16 Ø 6 mm × 0.16 Ø	788 197 315 0.22 ----- 2–2.5% 2–2.5%
Teng et al. [45]	Cement Fly ash (Class-C) Silica fume Welan gun powder Steel fiber W/C	642–662 401–413 41–42 0.18–0.27% 1–3% 0.2
Ibrahim et al. [46]	Cement Fine sand Silica fume Ground silica HRWRA Steel fiber W/C	712 1020 231 161.5 31 0–2% 0.18–0.24
Azmee et al. [32]	Cement Silica fume Fly ash Sand Steel fiber W/C	360–900 90 or (10%) 270–450 or (30, 40, 50%) 620 1% 0.16
Yu et al. [47]	Cement Fly ash GGBS Limestone powder Sand Micro-sand Nano-silica Steel fiber W/C	582.1–896.3 259.9–267.9 266.1–274.5 264.6–272.9 1039.5–1106.6 216.6–230.5 24.3–25.8 0 0.16–0.2
He et al. [48]	Cement Silica fume Quartz powder Quartz sand-I Quartz sand-II W/C Superplasticizers Glass fiber High-performance polypropylene fiber	750 90 263 306 714 0.255 12 0–2 0–2
Chen et al. [49]	Cement Silica fume Ultra-fine silica powder Viscous agent Steel fiber Polypropylene fiber W/C Superplasticizers	737–1005 0–191 0–31 1 0–0.8 0–78 0.195 25
Fadzil et al. [50]	Cement Metakaolin Nano metakaolin Superplasticizers W/C	720 and 800 80 0–72 16 0.2

**Table 3:** Summary of fresh properties of UHPC.

Authors/ Reference	Material	W/C	SP	Fiber (%)	Slump (mm)	Spread (mm)	Air Content (%)
Wu et al. [27]	Silica fume	0.18	Polycarboxylate	Straight 0 1 2 3 Corrugated 1 2 3 Hooked 1 2 3	------- 215 190 165 138 ------- 178 153 123 ------ 179 139 104	-------	-------
Wang et al. [53]	SF 10% GGBS 0 20 40 LP 0 20 40	0.18	Amino sulfonic acid	NA	245 255 210 ----- 210 285 287	570 565 490 ----- 445 685 690	-------
Hung et al. [54]	SF + QP	0.135	Polycarboxylate	Macro-steel fiber 0 1 2	135 265 330	410 645 740	----
Meng et al. [30]	GNPs, SF, FA	0.2	Polycarboxylate	%GNP’s/%CNF’s 0/0 0.05/0.05 0.1/0.1 0.15/0.15 0.2/0.2 0.3/0.3	-----	-----	GNP’s/CNF’s 2.5/2.5 2.61/2.62 2.5/2.5 2.6/2.8 2.98/3.01 2.82/3.20
Mo et al. [55]	LS-30% MK 0 5 10 15 20	0.2	Polycarboxylate	NA	-----	296 287 274 267 248	7.89 7.57 7.42 7.30 7.67
Teng et al. [45]	Class-C fly ash 40% Silica fume 5% Air-detraining admixture, polyether, 0.8%	0.2	Polycarboxylate	WG 0% 0.18% 0.22% 0.27%	280 270 265 260	Mini V-funnel Flow Tim-Sec	1 1.51 2.51 3.03
						11 20 32 60	
Chen et al. [49]	Silica fume-SF Ultra-fine silicon powder-SFP	0.195	Polycarboxylate	(StF + PPF + SF + SFP) %	ID	262 255 257 ---- 260 255 250	690 530 540 ---- 490 530 510
				0 + 0 + 0 + 0 0.5 + 0.03 + 17.4 + 2.2 1 + 0.06 + 18.7 + 2.2 ----------------- 0.75 + 0.03 + 18.7 + 3.2 0.75 + 0.09 + 20 + 2.2 1 + 0.03 + 20 + 2.7	UHPC1 UHPC2 UHPC3 --------- UHPC4 UHPC5 UHPC6		
Yu et al. [51]	Nano-silica (%) 0 1 2 3 4 5	0.4	Polycarboxylic ether	Macro-steel fiber (0–2.5)%	337 331 315 284 263 225	-----	2 2.1 2.3 2.4 2.8 3.4
Christ et al. [35]	Fly ash (45%) silica fume (90%)	0.45	Polycarboxylate	St. F = 3%	PPF = 3%	210 216 219 218 220 221 240	-----
				0% 50% 60% 705 80% 90% 100%	100% 50% 40% 30% 20% 10% 0%		
Li et al. [56]	-LS = 20% and SF = 10% -Steel slag powder (SSP) -Hybrid magnesia expansive agent (EA)	0.16	Polycarboxylate	Straight steel fibers 2%			-----
					-----	610 610 605 600 590 585 560	
	SSP%	EA%					
	0 10 15 20 0 15 15	0 0 0 0 5 5 8					

## Data Availability

All the data available in manuscript.
